# Gene Signatures of T-Cell Activation Can Serve as Predictors of Functionality for SARS-CoV-2-Specific T-Cell Receptors

**DOI:** 10.3390/vaccines10101617

**Published:** 2022-09-27

**Authors:** Laura M. Mateyka, Philipp M. Strobl, Sebastian Jarosch, Sebastian J. C. Scheu, Dirk H. Busch, Elvira D’Ippolito

**Affiliations:** 1 Institute for Medical Microbiology, Immunology and Hygiene, Technical University of Munich (TUM), 81675 Munich, Germany; 2 German Center for Infection Research (DZIF), Partner Site Munich, 81675 Munich, Germany

**Keywords:** SARS-CoV-2 TCRs, TCR functionality prediction, scRNA sequencing

## Abstract

The importance of T cells in controlling SARS-CoV-2 infections has been demonstrated widely, but insights into the quality of these responses are still limited due to technical challenges. Indeed, understanding the functionality of the T-cell receptor (TCR) repertoire of a polyclonal antigen-specific population still requires the tedious work of T-cell cloning or TCR re-expression and subsequent characterization. In this work, we show that it is possible to discriminate highly functional and bystander TCRs based on gene signatures of T-cell activation induced by recent peptide stimulation. SARS-CoV-2-specific TCRs previously identified by cytokine release after peptide restimulation and subsequent single-cell RNA sequencing were re-expressed via CRISPR-Cas9-mediated gene editing into a Jurkat-based reporter cell line system suitable for high-throughput screening. We could observe differences in SARS-CoV-2 epitope recognition as well as a wide range of functional avidities. By correlating these *in vitro* TCR engineered functional data with the transcriptomic profiles of the corresponding TCR-expressing parental T cells, we could validate that gene signatures of recent T-cell activation accurately identify and predict truly SARS-CoV-2-specific TCRs. In summary, this work paves the way for alternative approaches useful for the functional analysis of global antigen-specific TCR repertoires with largely improved throughput.

## 1. Introduction

Since the beginning of the Coronavirus Disease 2019 (COVID-19) pandemic in December 2019 [1,2], insights into the establishment of the adaptive immunity against the Severe Acute Respiratory Syndrome Coronavirus Type 2 (SARS-CoV-2) were gained and exploited therapeutically with an unprecedented speed.

Such global efforts resulted in the quick approval of effective vaccines [3,4,5]. However, the rapid emergence of variants, in particular the Omicron strain (B.1.1.529) subvariants BA.1.1 and BA.2, have resulted in countless breakthrough infections in vaccinated individuals [6] partially as a result of neutralizing antibody escape [7,8,9,10], whereas T-cell immunity seemed globally preserved [11]. Alarmingly, a recent study showed a complete loss of neutralization activity against Omicron variants *in vitro* for some therapeutic antibodies, thus legitimately raising concerns about their clinical relevance [12]. Overall, despite the typical mild course of disease and the reduced risk of hospitalization after Omicron infection, the rate of (re-)infections remains high, and some patients still experience severe COVID-19 [13,14]. Thus, new vaccines (also targeting proteins other than spike) as well as novel treatments for severe COVID-19 are needed.

The adoptive transfer of virus-specific T cells has been used successfully in immunocompromised patients to prevent life-threatening reactivations of latent viruses [15,16,17,18]. Following the same principle, first attempts showed that the administration of SARS-CoV-2-specific T cells from convalescent donors mediated effective viral control and protection against lung pathology after SARS-CoV-2 infection, even in the absence of neutralizing antibodies [19]. This work further supports the therapeutic potential of T cells and opens up the possibility of adoptive T-cell transfer also in severe COVID-19 cases. However, for a broader applicability, including the transfer of autologous T cells engineered with defined SARS-CoV-2-specific T-cell receptors (TCRs), it is necessary to achieve a more profound understanding of the quality of the SARS-CoV-2-specific T-cell immunity. This holds true also for the development of new vaccines.

SARS-CoV-2-specific T-cell responses have been extensively characterized quantitatively by the upregulation of activation markers (such as CD154 and 4-1BB) [20,21] or the release of effector cytokines (such as IFN-γ, TNF-α and Granzyme B) [22,23,24,25] after peptide restimulation. However, this approach does not provide detailed information on the quality of such responses. Indeed, discrimination between high and low functional T cells is often hindered by the relatively high peptide dose used for restimulation. Additionally, low-functional cross-reactive T cells could be engaged [26], thus biasing the interpretation of the detected response.

The functional characterization of the TCR repertoire of an antigen-specific population might represent a more precise approach to estimate the global functionality, and thereby the protective capacity, of the recruited T-cell immunity but is still technically tedious to determine. Complete TCR sequences are in the meantime easily accessible via single-cell next generation sequencing after isolation of antigen-specific T cells via, for example, peptide-Major Histocompatibility Complex (pMHC) multimer staining or activation markers/cytokines after stimulation. While contributions were made in the field to predict the likelihood of TCR peptide binding from its primary sequence [27,28,29,30,31], this is still not possible with the desired precision for TCR functionality. Thus, the follow-up analyses to confirm the functional avidity of identified TCRs still require the generation of T-cell clones or the re-expression of the transgenic receptor in a laborious, time-consuming characterization process, which is feasible only for a limited amount of TCRs in parallel.

In our previous work, we have shown that, in principle, it is possible to exploit transcriptional shifts induced by peptide restimulation to estimate the functionality of entire epitope-specific TCR repertoires [32]. Therefore, we have isolated SARS-CoV-2-specific CD8^+^ T cells from non-severe COVID-19 donors for several immunodominant viral epitopes according to cytokine release (IFN-γ) after peptide restimulation. By single-cell RNA sequencing (scRNA seq), we accessed in parallel the global transcriptomic as well as the TCR of each individual cell. The *in vitro* functional characterization of a small subset of these TCRs allowed to define gene signatures of recent activation capable of discriminating high and low functional TCRs and to demonstrate that highly functional TCRs are normally recruited into non-severe COVID-19. By extending on this work, we here validated the predictive capacity of defined signatures in a larger dataset of TCRs, which allowed a further refinement and optimization of the technology for following studies.

## 2. Materials and Methods

### 2.1. Cell Culture, Antibody and pMHC Multimer Staining

#### 2.1.1. Isolation of Human Primary T Cells and Cell Culture

Peripheral blood mononuclear cells (PBMCs) were isolated from fresh blood using gradient density centrifugation according to the manufacturer’s guidelines (Pancoll human). PBMCs were cultured in RPMI 1640 supplemented with 10% FCS, 0.025% L-glutamine, 0.1% HEPES, 0.001% gentamycin and 0.002% streptomycin (complete RPMI) at 1 × 10^6^ cells/mL. Every three days, 180 U/mL IL-2 were added to the cells. The A549 lung cancer cell line was cultured in DMEM supplemented with 10% FCS, 0.025% L-glutamine, 0.1% HEPES, 0.001% gentamycin and 0.002% streptomycin (complete DMEM), while the K562 and the Jurkat triple parameter reporter (J-TPR) cell lines were cultured in complete RPMI. All cells were cultured at 37 °C, 5% CO_2_ in a humidified incubator.

#### 2.1.2. Flow Cytometry-based Cell Sorting

For cell sorting, J-TPR cells were stained with the surface antibodies anti-hTCR-FITC (1:50) and anti-mTRBC-APC (1:100). PBMCs were additionally stained with anti-CD3-PE (1:100) and anti-CD8-eF450 (1:200). Surface staining was performed for 20 min on ice protected from light. Cells were washed twice and resuspended at a concentration of 20 × 10^6^ cells/mL in FACS buffer (PBS 1X, 0.5% bovine serum albumin, pH 7.45) supplemented with 2 mM EDTA. Immediately before sorting, propidium iodide (PI) was added (1:100) for live/dead discrimination. J-TPR cells were sorted for mTRBC^+^ and PBMC for CD3^+^ CD8^+^ mTRBC^+^ expression on the MoFlo Astrios EQ. Cells were taken into culture at a concentration of 1 × 10^6^ cells/mL. For sorted primary T cells, 180 U/mL IL-2 were added.

#### 2.1.3. pMHC Multimer and Surface Marker Stainings

pMHC monomers were produced in-house as previously described [33]. 0.4 μg biotinylated pMHC was multimerized on 0.1 μg fluorophore-conjugated Streptavidin backbone for 45 min in 10 µL multimerization volume and filled up to 50 μL after incubation with FACS buffer. 50 µL of fluorescently labelled multimers were then added to up to 5 × 10^6^ cells and incubated for 45 min on ice protected from light. After 25 min, surface antibody staining solution was added and incubated for the remaining 20 min of the multimer staining. Surface markers used for determining TCR knock-in efficiency and multimer staining were mTRBC-APC/Fire750 (1:50), mTRBC-APC (1:200), hTCR-FITC (1:50) and CD8-PB450 (1:200).

### 2.2. TCR Re-Expression by CRISPR-Cas9-Mediated OTR

#### 2.2.1. TCR DNA Template Design

DNA constructs for CRISPR-Cas9-mediated orthotopic TCR replacement (OTR) consisted of the following structure: 5′ homology arm (300–400 base pairs (bp)), P2A, TCR-β (including mTRBC with additional cysteine bridge [34]), T2A, TCR-α (including mTRAC with additional cysteine bridge [34]), bGHpA tail, 3′ homology arm (300–400 bp). All transgenic TCR constructs for integration at the TRAC locus were synthesized with Twist Bioscience and dsDNA was generated by PCR as previously described [35,36].

#### 2.2.2. T-Cell Activation for CRISPR-Cas9-Mediated OTR

For CRISPR-Cas9-mediated OTR in PBMCs, cells were activated for 48 h at 37 °C, 5% CO_2_ at a concentration of 1 × 10^6^ cells/mL in complete RPMI with the addition of 1.5 µL/1 × 10^6^ cells CD3/CD28 expamer [37], 300 U/mL IL-2, 5 ng/mL IL-7 and 5 ng/mL IL-15. After two days, the expamer stimulation was stopped by incubation with 1 mM D-Biotin in PBS for 20 min at RT. Cells were washed twice with PBS and one final time with complete RPMI medium before CRISPR engineering.

J-TPR cells were seeded at a concentration of 1 × 10^5^ cells/mL in complete RPMI medium 3 days before CRISPR-Cas9 experiment to ensure exponential growth conditions at the timepoint of editing.

#### 2.2.3. CRISPR-Cas9-Mediated OTR

Briefly, for the ribonucleoprotein (RNP) production, equal volumes of tracrRNA and crRNA (hTrac6 or hTrbc3) were mixed and annealed at 95 °C for 5 min. 20 µM electroporation enhancer (400 µM) was added to the gRNA (tracrRNA + crRNA). Cas9 (61 µM) was diluted to 6 µM with PBS. RNPs were generated by slowly adding equal volumes of 6 µM Cas9 to gRNA and incubating for 15 min at RT. For electroporation, hTRAC6 RNP, hTRBC3 RNP, and the template DNA (1 µg/µL) were incubated for 30 s (for 1 × 10^6^ cells, 1 µL each). Cells were resuspended in SE buffer (J-TPR cells) or P3 buffer (PBMCs) with added supplement (18 µL/100 µL) and subsequently mixed with assembled RNPs for nucleofection (EH-100 program). J-TPR cells were transferred in U-bottom plates with 30 µM HDR enhancer for 18 h overnight. The maximum HDR enhancer concentration used was based on the manufacturer’s suggestions regarding toxicity. The enhancer was removed the following day by centrifugation of cells and resuspension in fresh medium. PBMCs were cultivated in complete RPMI medium at 1 × 10^6^ cells/mL supplemented with 180 U/mL IL-2.

### 2.3. TCR Functional Validation

#### 2.3.1. Antigen-Specific Activation of TCR-Engineered J-TPR Cells

K562 leukemic cells, retrovirally transduced with the appropriate HLA class I molecule, were irradiated at 80 Gy. After irradiation, cells were incubated with a log-scale peptide titration (10^−12^ M–10^−4^ M) and incubated overnight at 37 °C, 5% CO_2_ at a density of 1.5 × 10^6^ cells/mL. On the following day, peptide-pulsed cells were washed twice and co-incubated with TCR-engineered J-TPR-CD8^+^ cells at an E:T ratio of 1:1 (50,000 cells each) for 18 h. For the positive control, J-TPR cells were treated with 25 ng/mL PMA and 1 μg/mL Ionomycin. J-TPR cells without the addition of K562 cells served as negative control. After overnight incubation, J-TPR cells were stained for surface markers with anti-mTRBC APCfire (1:50) for 20 min on ice in the dark and subsequently with PI (1:100) for live/dead discrimination before flow cytometry acquisition. Readouts of the J-TPR cell line activation were the fluorescence reporter molecules GFP and CFP that were coupled with the transcription factors NFAT and NF-kB, respectively.

#### 2.3.2. xCelligence Killing Assay

Two passages before the assay, medium condition of the A549 cells were changed from complete DMEM to complete RPMI as T cells are more sensitive to medium conditions. A549 cells, transduced with the appropriate HLA class I molecule, were pulsed with 10^−5^ M of the respective peptide for 2 h at RT at a cell concentration of 3 × 10^6^ cells/mL. Cells were washed twice and 1 × 10^4^ target cells/well were added to xCelligence^®^ plates. Plates were incubated for 24 h in the xCELLigence^®^ instrument at 37 °C, 5% CO_2_ and the cellular impedance was measured every 15 min. After 24 h, TCR-engineered CD8^+^ T cells specific for the respective epitope were added with a final concentration of 50 U/mL IL-2. For the positive control, 2% TritonX was added to the cells whereas peptide-pulsed A549 cells without the addition of CD8^+^ T cells and non-pulsed A549 cells with the addition of epitope-specific CD8^+^ T cells served as negative controls. The plates were then incubated for an additional 48 h in the xCelligence^®^ instrument at 37 °C, 5% CO_2_ with impedance measured every 15 min. The killing capacity of the cells was quantified by calculating the area under the curve (AUC) ratio of pulsed to unpulsed target cells. The ratio was then subtracted from 1 and normalized based on the AUC ratio for the positive control (100%) and the negative control (0%).

### 2.4. Analyses of Single-Cell RNA Sequencing Data

Single-cell RNA sequencing of sorted cells was performed with the 10x genomics technology. For details on the library preparation and generation of the dataset, as well as for cell filtering and definition of the original gene sets, refer to [32].

#### 2.4.1. Data Pre-Processing

To integrate the data sets from the two experiments, raw gene counts for the filtered cells were combined for all genes present in both experiments. Highly variable genes were identified, the number of counts, percentage of mitochondrial genes, and cell cycle score regressed out, and the variance was scaled to unit variance and zero mean. The merged data were batch-corrected using batch-balanced k nearest neighbors (bbknn) for the individual donors.

#### 2.4.2. Refinement of Gene Sets According to a Large Set of Characterized TCRs

Each highly variable gene was correlated via Pearson correlation to the functionality measured by NFAT EC_50_ values for the reactive TCRs and the slope, *p*-value, and r-value recorded for all significantly correlated genes. A functionality score was subsequently calculated for each cell on the n top genes (n = 2, 3, …, 101) of the correlating gene list sorted by r value. This allowed us to plot the correlation variables between each of these gene sets and the TCR functionality to facilitate the decision for the number of genes to be considered, maximizing the correlation with TCR functionality (Figure S5B). 

For reactivity scoring, Wilcoxon rank sum differential gene expression analysis was performed between reactive and non-reactive TCRs. The genes were sorted by the z-score for reactive TCRs, and a reactivity score was calculated for each cell on n top genes (n = 2, 3, …, 101). Kruskal H test statistics were used to decide on the number of genes resulting in the best differentiation between reactive and non-reactive TCRs (Figure S5A).

### 2.5. Quantification and Statistical Analysis

Data were displayed using GraphPad Prism (V9.1) or the seaborn (0.10.0) and matplotlib (3.1.3) packages in python. Statistical and correlation analyses were performed in GraphPad Prism and with stat.linregress method from the scipy (1.4.1) module, respectively. Significance is defined as not significant (ns), * *p*-value < 0.05, ** *p*-value < 0.01, *** *p*-value < 0.001, **** *p*-value < 0.0001. Information on statistical tests used for individual figures can be found in the figure legends.

## 3. Results

### 3.1. Generation of SARS-CoV-2-Specific J-TPR-CD8^+^ Cells

In our previous work, we identified TCRs specific for several SARS-CoV-2 epitopes by scRNA seq (Table S1). For two repertoires (A1/ORF3a_FTS and A3/ORF1_VTN epitopes), we functionally characterized eleven TCRs, and used the transcriptomic data of the TCR-expressing parental cells to define signatures containing genes differentially expressed between epitope-reactive and non-reactive TCRs (reactivity score) or genes that correlated with IFN-γ EC_50_ values (functionality score), which were obtained in TCR-engineered T cells. Based on these scores, we could discriminate between high and low functional TCRs for these two epitopes according to their gene expression and predict the functional landscape of the TCRs composing the other nine epitope-specific repertoires (Wagner et al., 2022).

In this study, we primarily aimed at validating the performance of the identified gene signatures in predicting TCR functionality. For this purpose, we selected for re-expression 30 TCRs specific to the nine SARS-CoV-2 epitopes that were not in-depth analyzed in the previous study (Table S2). To challenge the predictive scores, TCRs were selected to be spread across the functionality score/reactivity score landscape. We defined as “predicted functional” all TCRs with a positive functionality and reactivity score, whereas we assigned the label of “predicted low- to non-functional” to all TCRs with a negative functionality score, a negative reactivity score, or that showed negative values for both signatures (Table S3). For the evaluation of TCR functionality, we used the Jurkat-based triple parameter TCR signaling reporter (J-TPR) cell line. J-TPR cells are derived from the Jurkat E6.1 cell line, which is commonly used to investigate TCR functions. In comparison to primary T cells, J-TPR cells allow for higher comparability and throughput and enable easy readouts for functional TCR screening. Indeed, readout of TCR activation is achieved by the coupling of T-cell activation transcription factors (NFAT, NF-kB, AP-1) with fluorescence reporter molecules (GFP, CFP, mCherry, respectively) [38]. To study MHC class I-restricted TCRs, J-TPR cells transduced with the CD8αβ co-receptor (J-TPR-CD8^+^) were used. Coexpression of the CD8αβ co-receptor stabilizes the interaction between the TCR and the cognate pMHC, thereby increasing the sensitivity of TCR activation [39]. For easier detection of the transgenic TCR by flow cytometry, the TCR alpha and beta chains were engineered to contain a murine constant region [34].

**Figure 1:**
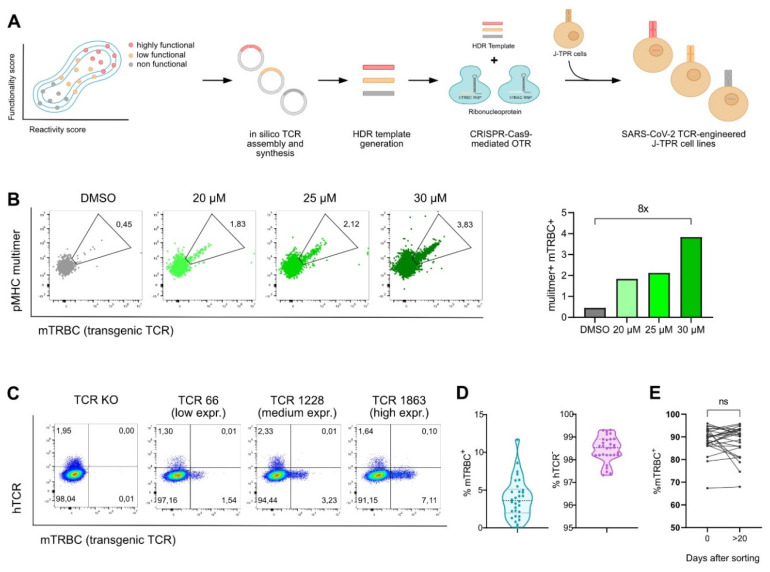
**Generation of SARS-CoV-2 TCR-engineered J-TPR-CD8^+^ cells.** (**A**) Schematic workflow describing the generation of SARS-CoV-2 TCR-engineered J-TPR-CD8^+^ cells. TCRs were selected based on their functionality and reactivity scores. Transgenic TCRs were in silico assembled, synthesized, and cloned into a vector. DNA templates were generated via PCR and eventually delivered into J-TPR-CD8^+^ cells together with TRAC and TRBC ribonucleoproteins (RNPs) for TCR replacement. (**B**) J-TPR-CD8^+^ cells were processed for CRISPR-Cas9 OTR as described in (**A**). HDR enhancer was added to the cell culture after electroporation for 18 h at the indicated concentrations. Shown are flow cytometry blots (left) and quantification (right) of multimer^+^ mTRBC^+^ cells (pregated on living lymphocytes). (**C**) Exemplary flow cytometry blots showing the endogenous TCR KO (hTCR^−^) and the transgenic TCR KI (mTRBC^+^) on day 4 post genome editing. Depicted are examples of low, medium, and high KI efficiencies. (**D**) Summary of the frequencies of mTRBC KI and hTCR KO of all SARS-CoV-2-specific TCRs 4 days post CRISPR-Cas9 editing depicted as violin plot. The mean is shown as wide dashed line and the quartiles are shown as tight dashed lines. (**E**) mTRBC expression on day 0 and day >20 after flow cytometry-based cell sorting. Data points per TCR are connected by a line. Statistical analysis was performed with a paired *t*-test. ns, *p* > 0.05.

After *in silico* assembly and synthesis, transgenic TCRs were integrated into J-TPR-CD8^+^ cells by CRISPR-Cas9-mediated orthotopic TCR replacement (OTR) (Figure 1A), a technology that enables the integration of the transgenic TCR into the endogenous TCR alpha locus by homology-directed repair (HDR), thus allowing for physiological regulation of TCR expression. The concomitant knock-out (KO) of the endogenous TCR beta locus eventually leads to the complete replacement of the endogenous TCR with the transgenic TCR [36,40]. In comparison to CRISPR-Cas9 OTR in primary T cells (Moosmann et al., 2022), integration of a transgenic TCR via OTR in the J-TPR-CD8^+^ cell line occurred at very low efficiencies. Therefore, we optimized our existing protocol by adding a HDR enhancer after electroporation to the culture for 18 h, which resulted in improved knock-in (KI) frequencies up to around eight-fold (Figure 1B). To generate SARS-CoV-2 TCR-engineered J-TPR-CD8^+^ cells, we thus added 30 µM HDR enhancer for CRISPR-Cas9 editing.

For all 30 TCRs, we observed transgenic TCR KI efficiencies of 3.92% ± 2.61 (0.09–11.7%), and endogenous TCR KO rates of 98.46% ± 0.56 (97.32–99.31%) (Figure 1C,D and Figure S1A). Post CRISPR editing, SARS-CoV-2 TCR-expressing J-TPR-CD8^+^ cells were purity sorted for hTCR^−^ mTRBC^+^ (Figure S1B). Importantly, the expression of the transgenic TCRs did not decline but was rather stably maintained over time (Figure 1E).

### 3.2. Gene Signature Scores Predict Epitope Specificity

**Figure 2:**
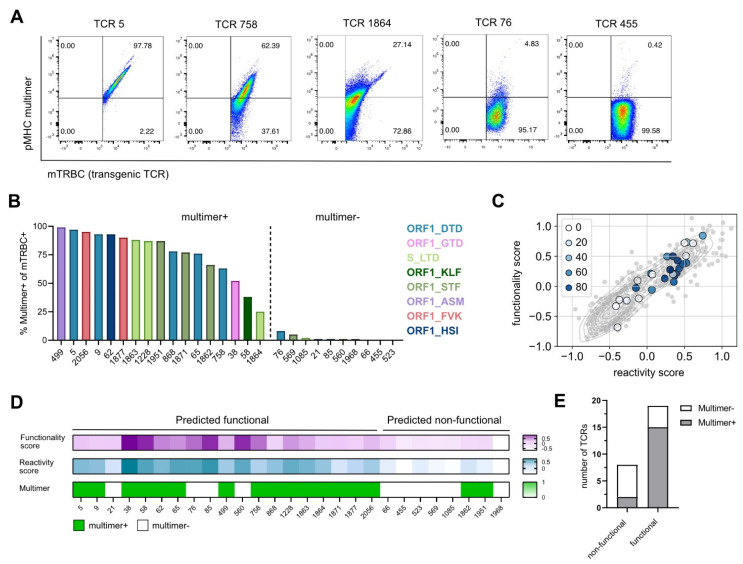
**Gene signature scores predict epitope specificity.** (**A**) Flow cytometry blots of TCRs with a high, medium, and low pMHC multimer staining recognizing the epitopes ORF1_DTD and S_LTD, pre-gated on mTRBC^+^ living J-TPR-CD8^+^ cells. (**B**) Bar graph showing the frequency of multimer^+^ mTRBC^+^ J-TPR-CD8^+^ cells arranged according to frequency. Multimer^+^ and multimer^−^ TCRs are delimited by a dashed line. TCRs recognizing the same epitope are depicted in the same color. (**C**) Gene signature scores overlapping with pMHC multimer staining. TCRs are color-coded according to the frequency of multimer^+^ J-TPR-CD8^+^ cells. (**D**) Heat map of functionality and reactivity scores depicted as a gradient, and pMHC multimer reactivity shown as positive or negative readout. (**E**) Quantification of non-functional and functional TCRs according to pMHC multimer staining.

**Figure 3:**
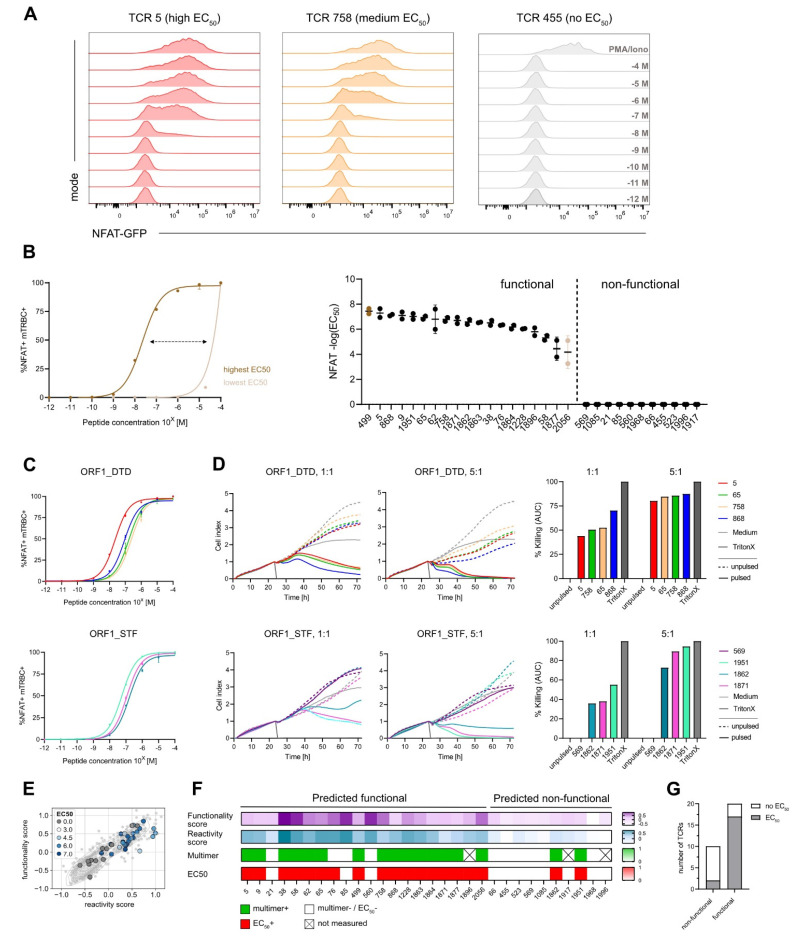
**Gene signature scores identify functional TCRs.** (**A**) Coculture assay of SARS-CoV-2-specific J-TPR-CD8^+^ cells with HLA-matched, peptide-pulsed K562 cells. MFI of NFAT signal according to peptide concentrations (10^−4^ M to 10^−12^ M) is shown representative of TCRs with high, medium, and low EC_50_ values of NFAT-GFP. (**B**) Range of NFAT EC_50_ values (left). Summary of NFAT EC_50_ values ordered according to frequency. Functional and non-functional TCRs are delimited by a dashed line (right). Highest and lowest EC_50_ value is colored dark brown and light brown, respectively. (**C**) Normalized mTRBC^+^ NFAT^+^ peptide titration curves of TCRs binding to epitopes ORF1_DTD (top) and ORF1_STF (bottom). (**D**) xCELLigence-based primary CD8^+^ T-cell killing assays with peptide-pulsed HLA-A*01 and HLA-A*11 A549 cells at E:T 1:1 and 5:1 for TCRs binding to epitopes ORF1_DTD and ORF1_STF (left). Quantification of killing as calculation of the area under the curve (right). (**E**) Gene signature scores overlapping with EC_50_ scores. TCRs are color-coded according to EC_50_ values. (**F**) Heat map of functionality and reactivity scores as a gradient, and pMHC multimer reactivity and measurable EC_50_ value defined as positive or negative. (**G**) Quantification of non-functional and functional TCRs according to EC_50_ values. AUC: Area under the curve.

We next evaluated the binding specificity of all re-expressed TCRs to their respective SARS-CoV-2 epitope, which we defined as a first pre-condition for a TCR to be functional, by performing pMHC multimer staining. For unbiased evaluation of multimer reactive cells on population purity, the frequency of multimer^+^ cells was calculated based on mTRBC expression (Figure 2A and Figure S2). TCRs with a frequency of multimer^+^ cells higher than 5% were considered specific for the epitope for which they were isolated, whereas frequencies of multimer^+^ cells lower than 5% indicated TCRs unspecific for SARS-CoV-2 epitopes (Figure 2B). Additionally, all TCRs classified as SARS-CoV-2-specific showed a positive correlation between the transgenic TCR expression and the multimer staining, which further supported the reliability of the applied cutoff (Figure 2A and Figure S2). For a few TCRs putatively specific for the epitope ORF1_VPF (TCR 1896, TCR 1917, TCR 1996), no multimer staining could be detected; as they showed high sensitivity to peptide stimulation in following experiments (see Figure 3), we questioned the functionality of the pMHC multimer and excluded these TCRs from further analyses.

To test if our previously identified gene signatures (functionality score, reactivity score) were able to predict epitope specificity, we overlaid the gene scores with the information about pMHC multimer staining. Interestingly, we observed a strong enrichment of truly SARS-CoV-2-specific TCRs in the gene score^high^ area (Figure 2C), which contains the predicted functional TCRs (Table S3). In more detail, we found that from 19 out of 27 TCRs that were predicted to be functional, 15 TCRs showed a positive pMHC multimer staining and thereby confirmed their epitope specificity. On the contrary, two out of the eight TCRs predicted as non-functional reacted to SARS-CoV-2 multimers after re-expression (Figure 2D,E). In summary, the defined gene signatures predicted TCR specificity with a sensitivity of 88% and a specificity of 60%.

### 3.3. Gene Signature Scores Identify Functional TCRs

To assess TCR functionality, firstly, we performed coculture assays of TCR-engineered J-TPR-CD8^+^ cells with peptide-pulsed HLA-matched K562 cells. An increase in NFAT-GFP producing cells could be seen according to both the peptide titration from 10^−12^ M to 10^−4^ M (Figure S3A) and the functionality of the TCR (Figure 3A). Indeed, highly functional TCRs showed higher sensitivity to less amount of peptide in comparison to low functional TCRs. We then quantified TCR functionality by the median effective concentration EC_50_. For visual comparison, NFAT-GFP^+^ frequencies are depicted as normalized or raw data plots (Figure S3B,C). We observed a wide functional heterogeneity, with log10 NFAT EC_50_ values ranging from −7.65 (TCR 499) to −3.2 (TCR 2056) for the 19 functional TCRs, while 11 TCRs did not show any reactivity (Figure 3B). The dynamics of NF-κB expression of J-TPR-CD8^+^ cells showed a trend similar to NFAT signals that resulted in a comparable ranking of EC_50_ values, as indicated by the strong correlation between NFAT and NF-κB EC_50_ values (Figure S4). This observation confirms that the measured EC_50_ values are reporter independent, as also previously described [39].

To confirm the functionality of our TCRs and the reliability of the NFAT EC_50_ values from the J-TPR system, we finally performed cytotoxicity assays using OTR-engineered primary CD8^+^ T cells and HLA-matched, peptide-pulsed A549 target cell lines. For re-expression, we selected TCRs specific for the epitopes ORF1_DTD and ORF1_STF as they were highly functional but also showed some variability in their EC_50_ values (Figure 3C). All four TCRs specific for the epitope ORF1_DTD showed efficient target cell killing at a high E:T ratio (Figure 3D, top). Interestingly, at a lower E:T ratio, where subtle functional heterogeneity could be better emphasized, we could observe differences with a hierarchy in line with the corresponding NFAT EC_50_ values, except for TCR 5. For TCRs binding to epitope ORF1_STF, TCR 569 did not show any killing as expected from the previous functional characterization, as also no EC_50_ value could be determined. For the remaining three TCRs, we again observed efficient killing of target cells, which further matched the functionality ranking of NFAT EC_50_ values at both high and low E:T ratios. (Figure 3D, bottom).

Similar to the analyses of pMHC multimer staining, we assessed the performance of the defined gene signatures in predicting TCR functionality. By combining the two gene scores with NFAT EC_50_ values, we observed that TCRs with high expression of both scores also have high EC_50_ values, except for the two non-reactive TCRs, TCR 85 and TCR 560 (Figure 3E). However, we did not observe a strong correlation between these variables, thus high and low functional TCRs could not be precisely discriminated. If we now quantify the predictive scores against the measured NFAT EC_50_ values, 20 out of 30 TCRs (67%) were predicted to be functional, while only 17 out of 30 (57%) had an actual measurable EC_50_ value (Figure 3F,G), which overall resulted in a sensitivity of 89%. Worth of note is the fact that most of the non-functional TCRs showed low gene signature scores and undetectable multimer staining, except for TCR 76 and neglecting the set of ORF1_VPF-specific TCRs for which pMHC multimer stainings were not available. Altogether, this supports the fact that these TCRs are not specific for the epitope under investigation and indicate a certain level of background noise (TCRs with low gene scores) or bystander activation (TCRs with high gene scores) within the approach. Intriguingly, TCR 76 showed high peptide sensitivity, indicating that the previously observed dim multimer staining was presumably imputable to a weak TCR expression rather than an unspecific background signal of the pMHC multimer. Within the group of TCRs predicted as non-functional, we again found two functional TCRs, in line with their robust pMHC multimer staining, which set the specificity rate to 73%.

### 3.4. Improvement of the Predictive Precision of Gene Scores

**Figure 4:**
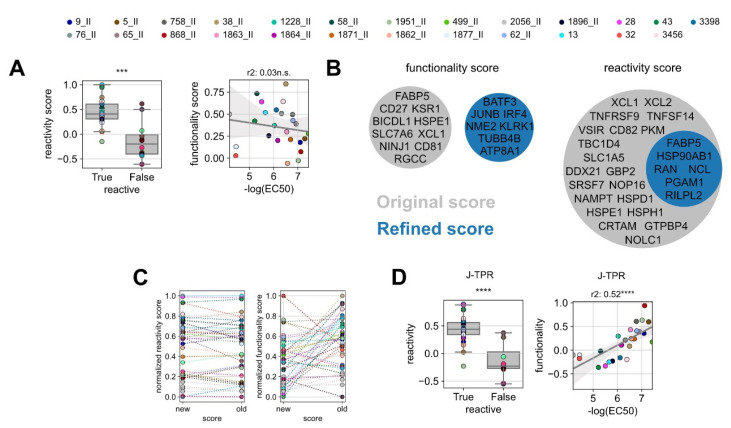
**Improvement of the predictive precision of gene scores.** (**A**) Box plot of the original reactivity score grouped into reactive and non-reactive TCRs (left) and linear regression analysis between *in vitro* functionality (NFAT EC_50_) and ‘original’ functionality gene score for reactive TCRs (right). (**B**) Venn diagrams of the genes defining the ‘original’ and ‘refined’ functionality and reactivity scores. (**C**) Comparison of the original and refined scores for reactivity (left) and functionality (right). Statistical testing was conducted by Kruskal–Wallis H test. (**D**) Box plot of the refined reactivity score grouped into reactive and non-reactive TCRs (left) and linear regression analysis between *in vitro* functionality (NFAT EC_50_) and ‘refined’ functionality gene score for reactive TCRs (right). Linear correlation analysis was performed by Pearson’s correlation. ns *p* > 0.05, *** *p* < 0.001, **** *p* < 0.0001.

The weak correlation between our previously identified gene signature scores and TCR functionality (Figure 3E) led us to hypothesize that the gene signatures defined on a certain experiment may not perform equally well on a new independent dataset, in particular if the training set is made of a limited number of TCRs and epitope specificities as in our case. To test this, we analyzed the performance of the reactivity and functionality scores separately. For the reactivity score, as it should indicate if a TCR is specific or not for a certain epitope, TCRs were simply defined as “True” or “False” whether they showed a measurable NFAT EC_50_ value or not. As expected, all but one of the functional TCRs showed positive reactivity score values, which were statistically higher than non-functional TCRs. For the functionality score, we could not observe a reliable correlation with the EC_50_ values (Figure 4A), thus indicating a certain degree of experimental dependency of this signature.

In the attempt of improving the predictive power of gene signatures, we *de novo* calculated the reactivity and functionality scores including all TCR:epitope pairs characterized so far (the pairs used for the training and the pairs used in this work as validation). For this, we redefined the gene list of the most differentially expressed genes between functional and non-functional TCRs, as well as the list of the top genes correlating to TCR functionality. From these lists, we identified the optimal number of top differentially expressed genes (‘refined’ reactivity score) and the optimal number of genes correlated to functionality (‘refined’ functionality score). The selection was carried out by iterative scoring of top n gene sets and score optimization by the selection of the most significantly discriminating of the best correlated gene set (Figure S5). Classical genes of effector T-cell functions were globally upregulated in most of the analyzed T cells; thereby, they were not enriched in any clonotype (Tables S4 and S5). Gene pools of the ‘refined’ functionality scores did not overlap with the original signature, while genes defining the ‘refined’ reactivity score were entailed in the ‘original’ signature (Figure 4B). In line with that, the individual reactivity scores of the TCRs remained stable after redefinition, whereas the values of the functionality scores changed drastically (Figure 4C).

We next applied the refined gene signatures to the whole TCR dataset. With the ‘refined’ reactivity score, the discrimination of functional and non-functional TCRs was overall similar to the one obtained with the original signature, in accordance with their degree of gene overlap, and it even improved by one level of significance (Figure 4D, left). Notably, the ‘refined’ functionality score sharply increased in its correlation with TCR functionality (Figure 4D, right) and allowed for more precise discrimination of low and high functional TCRs.

## 4. Discussion

For this study, SARS-CoV-2-specific CD8^+^ T cells were identified from mild convalescent COVID-19 donors. Similarly to the related SARS-CoV virus [41,42,43], the *ex vivo* frequency of SARS-CoV-2-specific CD8^+^ T cells is also very low. Therefore, for easier detection, we and others expanded SARS-CoV-2-specific CD8^+^ T cells with SARS-CoV-2 epitopes by short-term *in vitro* culture [22,23,24]. We then identified SARS-CoV-2-specific CD8^+^ T cells by restimulation with the respective epitope and flow cytometry-based sorting of IFN-γ expressing T cells, eventually followed by single-cell RNA sequencing [32].

By using scRNA seq, we accessed in parallel the transcriptomic and the TCR sequence of SARS-CoV-2 CD8^+^ T cells. In ‘hot’ environments, meaning in situations of *in vivo* antigen persistence such as cancer patients or active infections, this combined information suffices for the identification of antigen-specific T cells (and corresponding TCRs) directly *ex vivo* by gene signatures of recent T-cell activation [44,45]. However, in ‘cold’ environments (absence of antigen), the information on gene expression is only long-term and might not reflect actual functionality. Indeed, in a study comparing the reactivity between the TCR repertoires of peptide-responsive and pMHC multimer-binding T cells, T cells from these two experimental settings showed the same specificity but extensive differences in gene expression profiles [46]. For our study, SARS-CoV-2 TCRs were retrieved from recovered individuals who had been infected months before. Thus, the short peptide restimulation prior to sequencing allowed to recreate *in vitro* such a hot environment and exploit the antigen-induced transcriptional shifts for the identification and functional characterization of SARS-CoV-2-specific TCRs based on gene expression.

We used the so-called ‘reactivity’ score mainly to predict TCR epitope-specificity. For this reason, the score was defined by genes differentially expressed between epitope-reactive and bystander TCRs and robustly identified the truly antigen-specific TCRs. We observed a certain proportion of TCRs unrelated to SARS-CoV-2 within the pool of TCRs that were supposedly specific for the virus, as isolated based on IFN-γ expression after restimulation. While IFN-γ is a robust marker of T-cell activation [47,48], this observation highlights that unspecific TCRs could still be included in the analyses due to either a bystander activation during sample processing or background noise of the technology used for cell sorting (e.g., IFN-γ catch). Thus, considering other genes in addition to IFN-γ would ensure a more precise definition of the actual repertoire of an antigen-specific population.

Notably, the genes composing the reactivity score remained quite similar between two independent experiments (our previously published dataset and the one characterized in this work), presumably due to the relevance of these genes in T-cell functions, regardless of the antigen specificity or the strength of activation. The combined expression of TNFRSF9, XCL1, XCL2, and CRTAM genes was also identified by others as the most distinct marker for virus-specific T cells in Influenza Virus and Cytomegalovirus [46]. Moreover, other genes (FABP5, PGAM1, RAN) have been shown to play a role in the regulation of T cells. Ran was demonstrated to be involved in regulatory functions in T-cell activation in mice [49]. *In vivo* experiments with PGAM1-deficient mice showed mitigated T-cell-dependent immune responses and a critical role of Pgam1 in regulating glycolysis in activated T cells [50]. Finally, FABP5 has been identified to be an immunometabolic marker of CD137-enriched exhausted T cells, which have superior effector functions and proliferation capacity [51]. Altogether, this body of evidence supports a broader applicability of the defined reactivity signature also to other settings of T-cell activation.

In addition to an accurate prediction of epitope specificity, we also aimed at identifying gene signatures informative of TCR functionality. For this purpose, we established a score defined by the genes best correlating with the NFAT EC_50_ values of TCR-engineered T cells. Differently from the reactivity score, the functionality score defined on a previous dataset could discriminate functional from non-functional TCRs but not high from low avidity TCRs on a new dataset, which stemmed from a poor overlap of genes composing the functionality score in the two settings (‘original’ versus ‘refined’ scores). One possible explanation could be the high peptide concentration (10^−6^ M) used for restimulation of SARS-CoV-2-specific TCRs prior to sequencing, which might flatten differences in gene expression profiles between low and high functional TCRs and thereby introduce some stochasticity in the genes resulting as differentially expressed. Moreover, the use of a limited number of TCRs in the ‘training’ set might have resulted in an experiment-dependent gene signature. Despite this limitation, we could still show that it is possible to obtain a gene signature strongly correlating with TCR functionality for a specific experiment upon characterization of a small set of TCRs. This can allow to infer from a limited number of characterized TCRs the functionality of the remaining repertoire(s), which might range from a few dozen up to hundreds of TCRs.

Besides the validation of gene signatures, we could also confirm the reliability of the Jurkat-based reporter cell line in recapitulating the functionality of primary TCR-engineered T cells [39]. This system allowed an easier TCR characterization and a faster identification of potential candidates for clinical application using autologous SARS-CoV-2 TCR-engineered T cells. Furthermore, we optimized the OTR protocol for Jurkat cells. Additional improvements are expected in light of the recent developments in CRISPR-Cas9-mediated transgene knock-in [52,53].

### Limitations of the Study

The majority of TCRs characterized in this study were highly functional. Ideally, more low-functional TCRs should have been included to further improve the discrimination of genes that are differentially expressed in high and low functional TCRs. However, the dependency on *in vitro* expansion to identify robustly antigen-specific CD8^+^ T-cell populations might represent a bias towards detection of highly functional TCRs. We are currently working on the development of TCR identification protocols without *in vitro* expansion.

## 5. Conclusions

Our data confirmed the potential of gene scores of recent T-cell activation in predicting TCR features. With a high prediction accuracy, we were thus able to identify the truly antigen-reactive and functional TCRs within SARS-CoV-2-specific polyclonal CD8^+^ T-cell populations. Predicting functional differences of TCRs, however, is more challenging and requires more research. We envision that with the here-described usage of epitope-induced T-cell gene signatures, we provide a first step towards predicting TCR functionality without the need for additional cloning, TCR sequencing, re-expression, and subsequent experimental fine-characterization. This would allow to understand the global functionality of polyclonal antigen-specific TCR repertoires with a remarkably higher throughput. This approach can be immediately applied to different scenarios, such as severe COVID-19 patients as well as vaccinated individuals, for which the quality of T-cell responses has not been fully characterized. Unraveling these questions would clarify whether any feature of the SARS-CoV-2-specific T-cell responses might associate with disease protection. Eventually, gene signatures might be implemented diagnostically to predict a disease state or outcome. Finally, our workflow of TCR identification and characterization can also be applied to other SARS-CoV-2 epitopes or other pathogens or T-cell antigens.

## Data Availability

Not applicable.

## Supplementary Materials

The following supporting information can be downloaded at: https://www.mdpi.com/article/10.3390/vaccines10101617/s1, Figure S1: Gating strategies for flow cytometry-based cell staining and sorting for the generation of SARS-CoV-2-specific TCR-engineered J-TPR-CD8^+^ cells. Related to Figure 1. (A) Exemplary gating strategy for TCR KI/KO staining on day 4 after CRISPR-Cas9 editing. (B) Exemplary sort raw data for TCR 5; Figure S2: pMHC multimer staining of SARS-CoV-2 TCR-engineered J-TPR-CD8^+^ cells. Related to Figure 2. (A) Exemplary gating strategy for the quantification of the frequency of multimer positive cells. (B) Flow cytometry plots of multimer^+^ mTRBC^+^ for all re-expressed TCRs. TCR-engineered cells were stained with their respective pMHC multimer; Figure S3: NFAT EC_50_ *in vitro* functionality of SARS-CoV-2-specific TCRs. Related to Figure 3. (A) Flow cytometry plots depicting the frequency of NFAT-expressing cells according to peptide titration from 10^−12^ M to 10^−4^ M. (B,C) Normalized (B) and raw (C) NFAT EC_50_ curves of all TCRs. TCRs specific for the same epitope are shown in the same graph; Figure S4: NF-κB EC_50_ *in vitro* functionality of SARS-CoV-2-specific TCRs. Related to Figure 3. (A,B) Normalized (A) and raw (B) NF-κB EC_50_ curves of all TCRs. TCRs specific for the same epitope are shown in the same graph. (C) Summary of NF-κB EC_50_ values. (D) Pearson correlation between NFAT and NF-κB EC_50_ values; Figure S5: Refinement of gene sets according to a large set of characterized TCRs. Related to Figure 4. (A) Mean difference (top), H statistic (middle) and *p*-value (bottom) are plotted for the Kruskal H test comparing the score of the n top genes from the differential expression between reactive and non-reactive TCRs. (B) Slope (top) r-value (middle), and *p*-value (bottom) are plotted for the Pearson correlation of the score of the top n genes for single gene correlation with TCR functionality. (C) Volcano plot for the differential gene expression analysis between reactive and non-reactive TCRs. (D) Volcano plot for the Pearson correlation of individual genes with the functionality of reactive TCRs; Table S1: List of SARS-CoV-2-derived 9-mer epitopes; Table S2: SARS-CoV-2-specific TCR sequences; Table S3: Classification of SARS-CoV-2-specific TCRs according to the predicted functionality; Table S4: Differentially expressed genes according to reactive and non-reactive clonotypes; Table S5: Correlation data for highly variable genes with TCR functionality.
