# Supplementary material for: Gene Signatures of T-Cell Activation Can Serve as Predictors of Functionality for SARS-CoV-2-Specific T-Cell Receptors
Source: Vaccines (Basel). 2022 Sep 27;10(10):1617. doi: 10.3390/vaccines10101617 (PMC9611811; doi:10.3390/vaccines10101617)
Supplement: Supplementary file 1 [file vaccines-10-01617-s001.zip › Key Resource Table_Mateyka et al.pdf]

| REAGENT or RESOURCE | SOURCE | IDENTIFIER |
|---|---|---|
| Antibodies | | |
| CD3-Pacific Blue (UCHT1) (1:100) | BD Biosciences | Cat#: 558117 |
| CD3-APC (UCHT1) (1:200) | Life Technologies | Cat#: 17-0038-42 |
| CD8-APC (RPA-T8) (1:400) | BioLegend | Cat#: 301049 |
| CD8-FITC (B9.11) (1:100) | Beckman Coulter | Cat#: A07756 |
| CD8a-PE (C8/144b) (1:100) | eBioscience | Cat#: 12-0086-42 |
| CD8a-PECy7 (SFCI21Thy2D3) (1:200) | Beckman Coulter | Cat#: 737661 |
| CD8a-APC-eFluor 780 (OKT-8) (1:100) | eBioscience | Cat#: 47-0086-42 |
| CD45-ECD (J33) (1:50) | Beckman Coulter | Cat#: A07784 |
| CD45-Pacific Blue (T29/33) (1:50) | DAKO | Cat#: PB986 |
| CD45-Pacific Orange (HI30) (1:50) | Exbio | Cat#: PO-684-T100 |
| hTCR α/β-FITC (IP26) (1:50) | BioLegend | Cat#: 306706 |
| mTRBC ß chain-APC (H57-597) (1:200) | BioLegend | Cat#: 109212 |
| mTRBC-APC/Fire750 (H57-597) (1:50) | Biolegend | Cat#: 109246 |
| Streptavidin-APC | eBioscience | Cat#: 17-4317-82 |
| Bacterial and virus strains | | |
| Stbl3 | Thermo Fisher Scientific | Cat#: C737303 |
| Biological samples | | |
| Human blood/PBMCs | Healthy volunteers | Institute for Medical Microbiology, Immunology and Hygiene, Technical University Munich |
| Chemicals, peptides, and recombinant proteins | | |
| Alt-R® Cas9 Electroporation Enhancer | IDT | Cat#: 1075916 |
| Alt-R® Cas9 HDR Enhancer | IDT | Cat#: 1081072 |
| Alt-R® S.p. HiFi Cas9 Nuclease V3 | IDT | Cat#: 1081061 |
| Ampicillin | Roth | Cat#: K029.1 |
| Biotin | IBA Lifesciences | Cat#: 6-6996-001 |
| DBCO-PEG4-Biotin | Jena Bioscience | Cat#: CLK-A105P4-10 |

| | | |
|---|---|---|
| Dulbecco's Modified Eagle's Medium (DMEM) | Life Technologies | Cat#: 10938025 |
| DMSO | Merck | Cat#: D8418 |
| DNA LoBind tubes | Sigma | Cat#: EP0030108051, EP0030108078, EP0030124359 |
| Dulbecco's Phosphate Buffered Saline (PBS) | Sigma-Aldrich | Cat#: D8537-500ML |
| EDTA | Roth | Cat#: 8040.2 |
| Fetal calf serum | Biochrom | N/A |
| HEPES | Life Technologies | Cat#: 15630056 |
| Herculase II Fusion DNA Polymerase | Agilent Technologies | Cat#: 600679 |
| Human serum | In house | N/A |
| Ionomycin | Sigma | Cat#: I9657 |
| LB-medium / agar | In house | N/A |
| L-Glutamine | Sigma | Cat#: G8540-100G |
| Pancoll human (Ficoll) (1.077g/ml) | PAN Biotech | Cat#: P04-601000 |
| Penicillin/Streptomycin | Life Technologies | Cat#: 10378016 |
| Phorbol myristate acetate (PMA) | Sigma | Cat#: P1585 |
| Propidium Iodide (PI) | Life Technologies | Cat#: P1304MP |
| Recombinant human IL-15 | Peprotech | Cat#: 200-15 |
| Recombinant human IL-2 | Peprotech | Cat#: 200-02 |
| Recombinant human IL-7 | Peprotech | Cat#: 200-07 |
| RPMI 1640 Gibco | Sigma-Aldrich | Cat#: R0883 |
| SARS-CoV-2 individual peptides | IBA | N/A |
| SARS-CoV-2 individual peptides | peptides & elephants | N/A |
| Triton X-100 | Sigma-Aldrich | Cat#: X100-500ML |
| β-mercaptoethanol | Life Technologies | Cat#: 31350010 |
| Critical commercial assays | | |
| Agencout AMPure XP | Beckman Coulter | Cat#: A63881 |
| High sensitivity DNA Kit | Agilent | Cat#: 5067-4626 |

| | | |
|---|---|---|
| P3 Primary Cell Kit | Lonza | Cat#: V4XP-3024 V4XP-3096 |
| Deposited data | | |
| Raw and analyzed ScRNA seq data | Wagner et al., 2022 | GEO: GSE190839 |
| SARS-COV-2 (Wuhan-Hu-1) | NCBI | NC_045512 |
| Experimental models: Cell lines | | |
| A549-ACE2-RFP-HLA-A*01:01 | In house production | N/A |
| A549-ACE2-RFP-HLA-A*11:01 | In house production | N/A |
| K562-HLA-A*01:01 BFP | In house production | N/A |
| K562-HLA-A*02:01 BFP | In house production | N/A |
| K562-HLA-A*03:01 BFP | In house production | N/A |
| K562-HLA-A*11:01 BFP | In house production | N/A |
| K562-HLA-B*08:01 BFP | In house production | N/A |
| K562-HLA-B*35:01 BFP | In house production | N/A |
| Experimental models: Organisms/strains | | |
| N/A | N/A | N/A |
| Oligonucleotides | | |
| Alt-R® CRISPR-Cas9 crRNA 5′-AGAGTCTCTCAGCTGGTACA-3′ for TRAC | IDT DNA | N/A |
| Alt-R® CRISPR-Cas9 crRNA 5′-GGAGAATGACGAGTGGACCC-3′ for TRBC | IDT DNA | N/A |
| Alt-R® CRISPR-Cas9 tracrRNA | IDT | Cat#: 1072532 |
| HDR template PCR primer fwd 5′-CTGCCTTTACTCTGCCAGAG-3′ | Merck | N/A |
| HDR template PCR primer rev 5′- CATCATTGACCAGAGCTCTG-3′ | Merck | N/A |

| Software and algorithms | | |
|---|---|---|
| Affinity Designer 1.9 | Serif | https://affinity.serif.com/ |
| Cell Ranger 3.0.2/5.0.0 | 10X genomics | https://support.10xgenomics.com/single-cell-gene-expression/software/pipelines/latest/installation |
| FlowJo V10 | FlowJo LLC | https://www.flowjo.com |
| GraphPad Prism 9 | Graphpad | https://www.graphpad.com |
| IncuCyte® S3 Software | Satorius | https://www.sartorius.com |
| Microsoft Excel | Microsoft | N/A |
| RTCA xCELLigence Software | Agilent | https://www.agilent.com |
| Scanpy 1.8.1 | Wolf et al., 2018 | https://doi.org/10.1186/s13059-017-1382-0 |
| Scirpy 0.10 | Sturm et al., 2020 | https://doi.org/10.1093/bioinformatics/btaa611 |
| Other | | |
| Äkta pureSuperdeso 200 10/300GL | GE | N/A |
| 4D-Nucleofector | Lonza | Cat#: AAF-1002B, AAF-1002X |
| CytoFlex S Cell Analyzer | Beckman Coulter | Cat#: |
| Incucyte® S3 Live-Cell Analysis System | Sartorius | Cat#: 4647 |
| MoFlo Astrios EQ | Beckman Coulter | B25982 |
| SimpliAmp Thermocycler | Applied Biosystems, Darmstadt, Germany | Cat#: A24811 |
| xCELLigence® RTCA eSight | Agilent | Cat#: 380601600 |
