# Supplementary figures and images for: Gene Signatures of T-Cell Activation Can Serve as Predictors of Functionality for SARS-CoV-2-Specific T-Cell Receptors
Source: Vaccines (Basel). 2022 Sep 27;10(10):1617. doi: 10.3390/vaccines10101617 (PMC9611811; doi:10.3390/vaccines10101617)

**A**

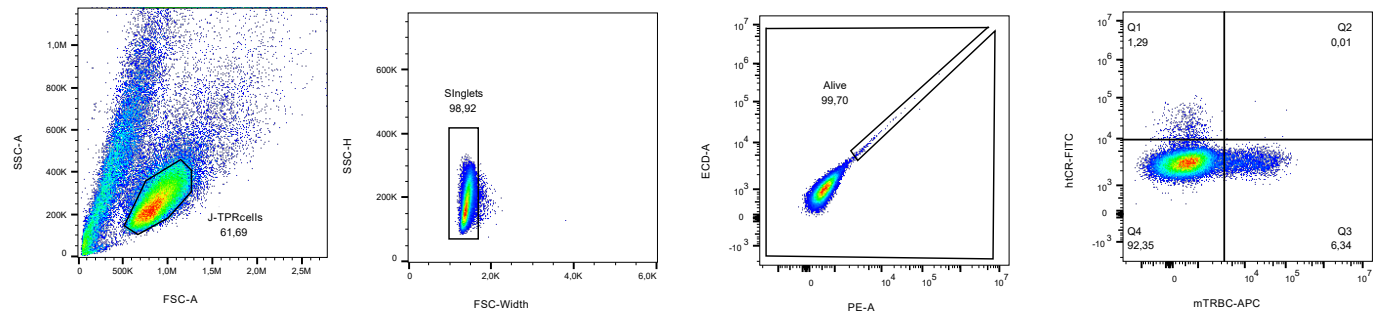

**B**

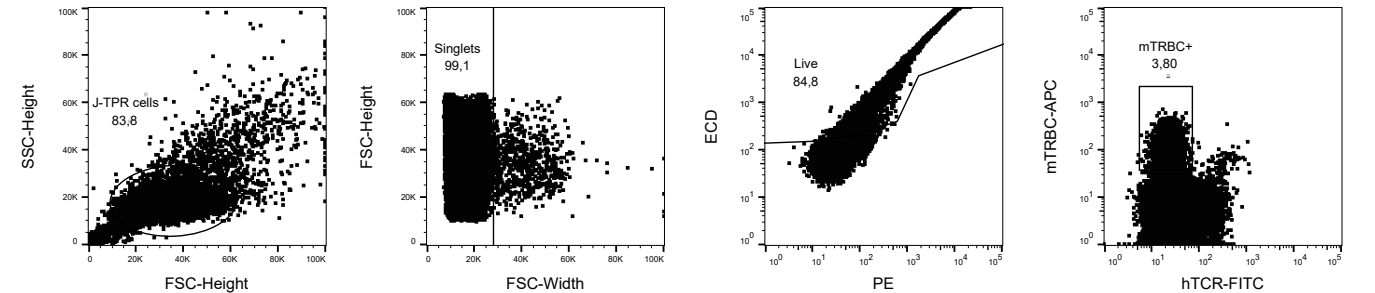

**Figure S1**

**A**

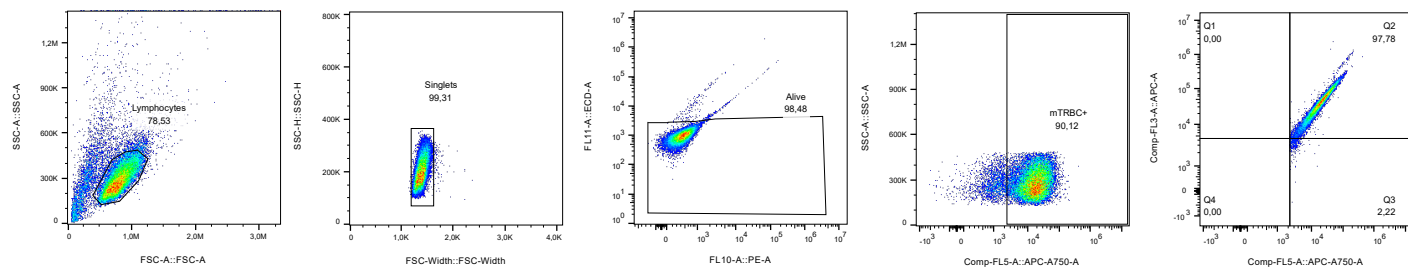

**B**

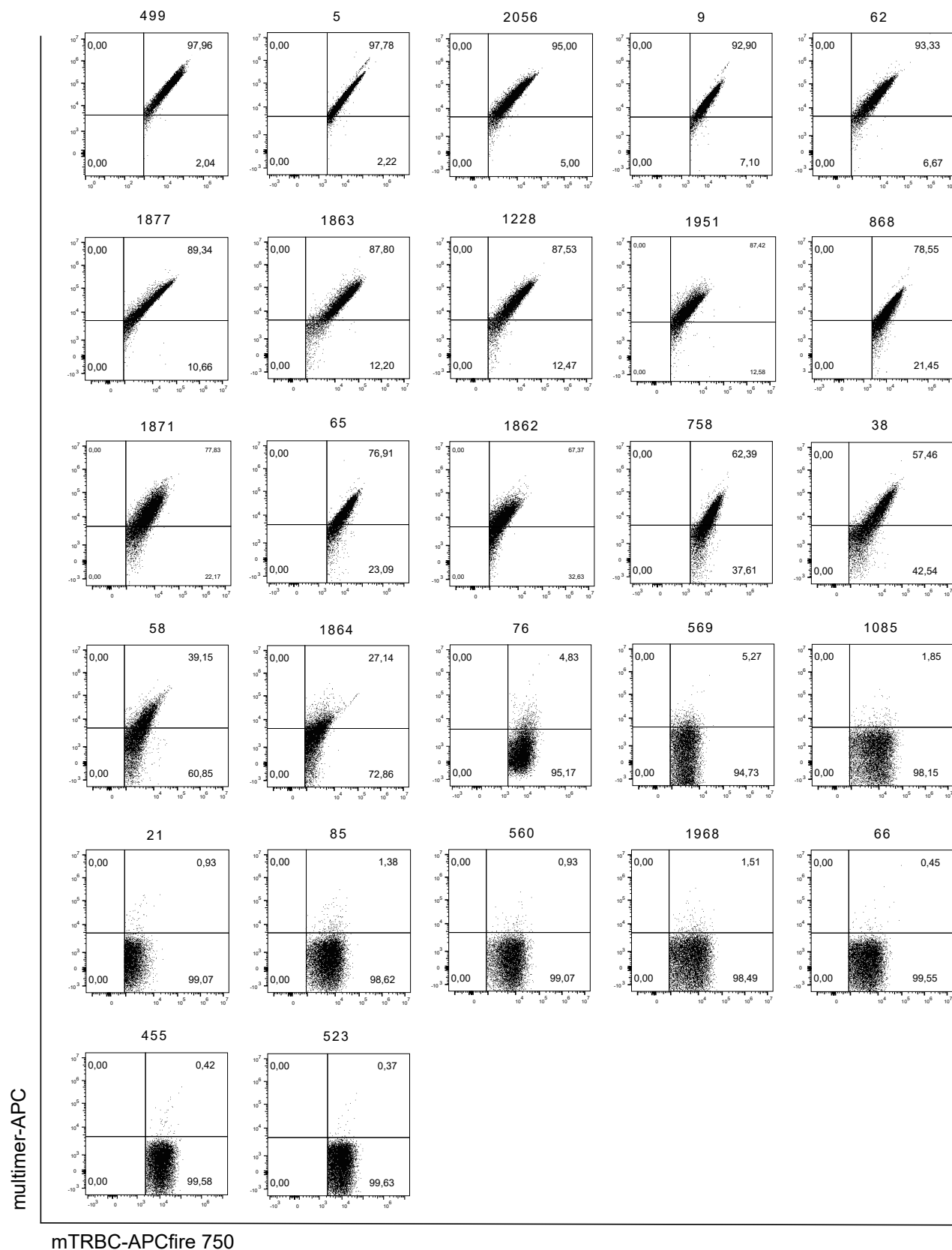

**Figure S2**

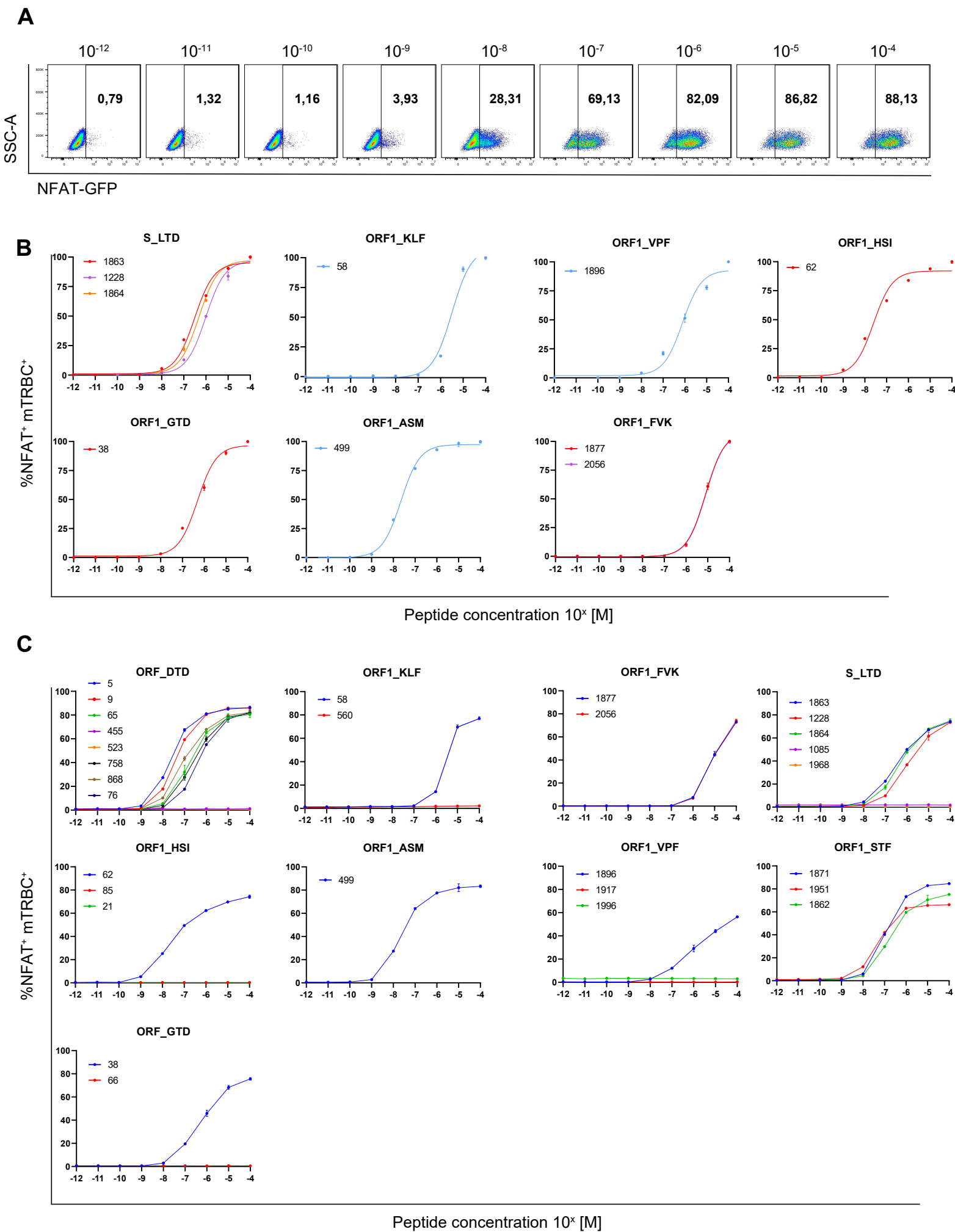

**Figure S3**

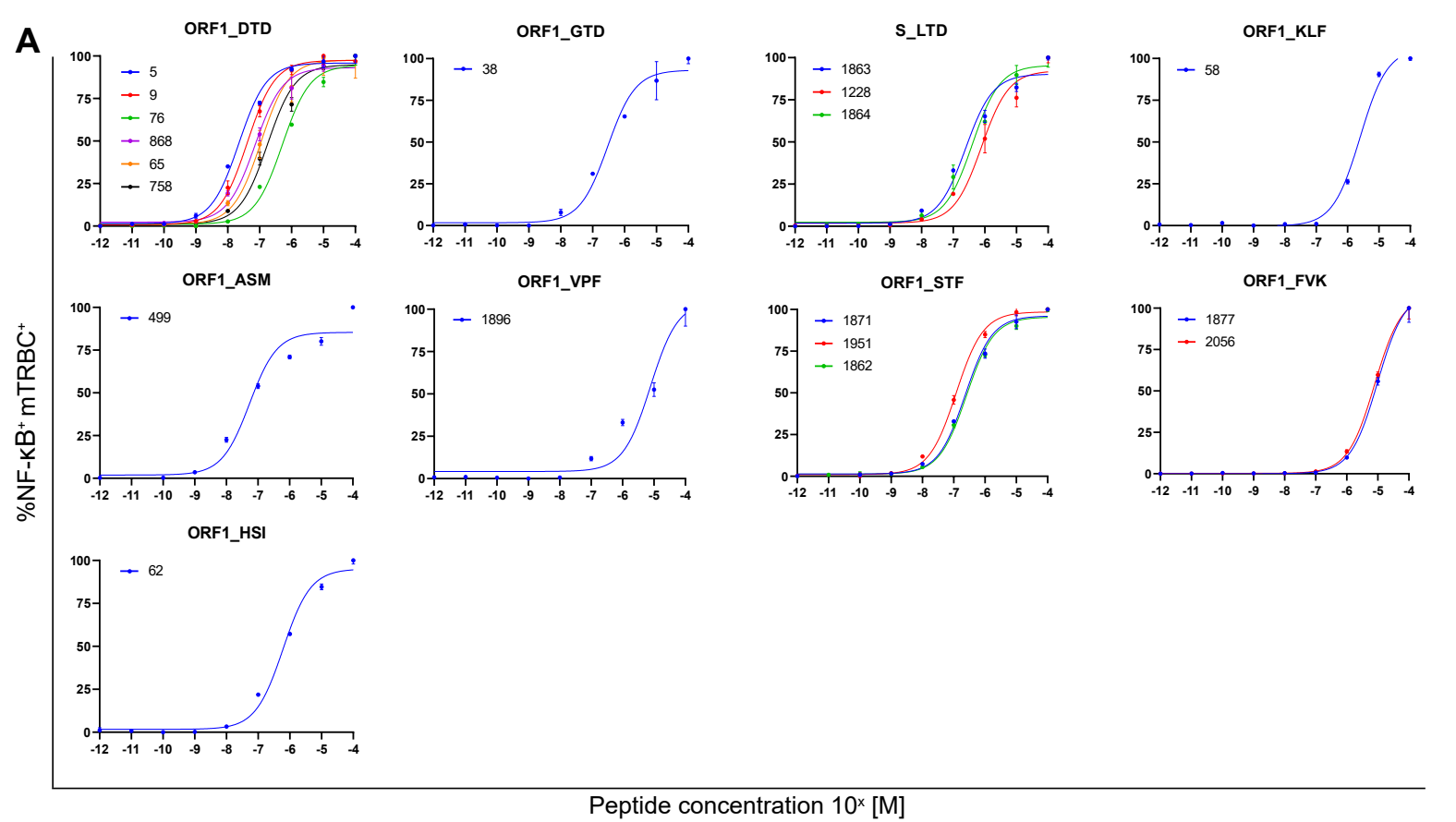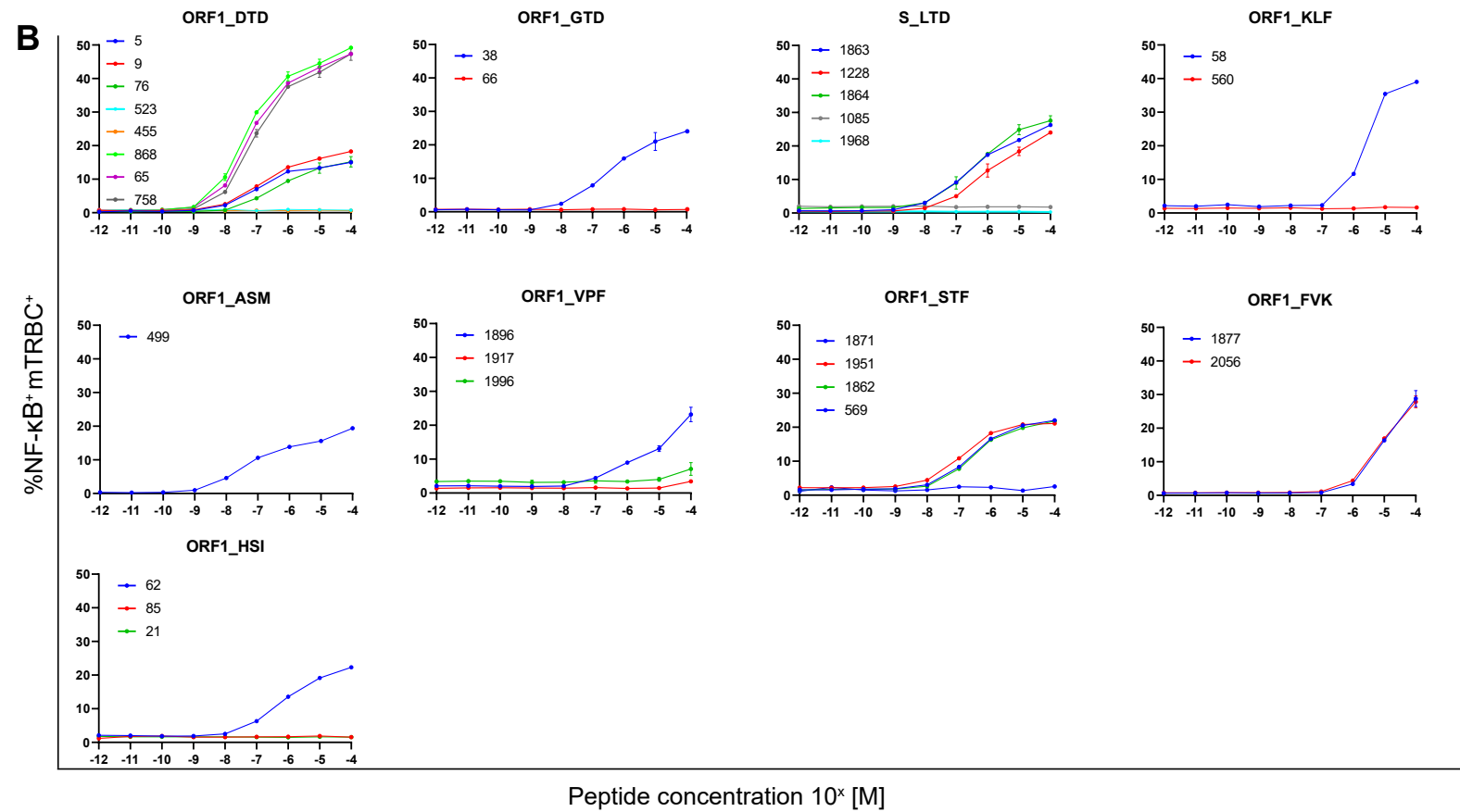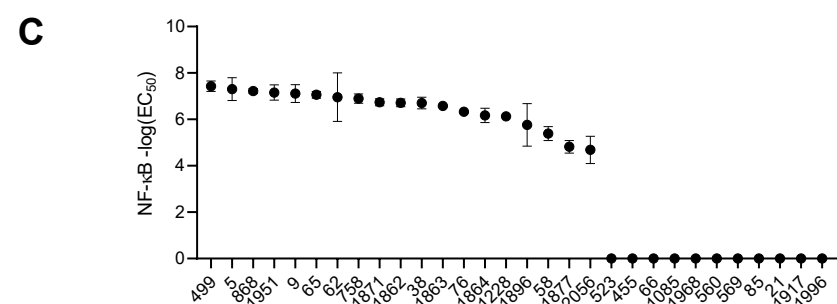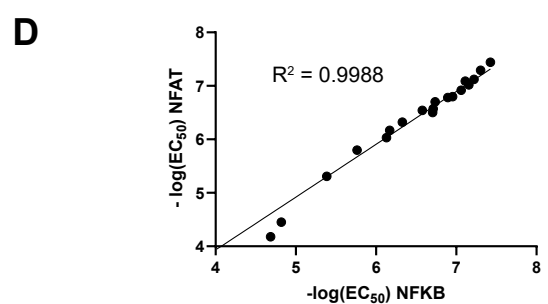

Figure S4

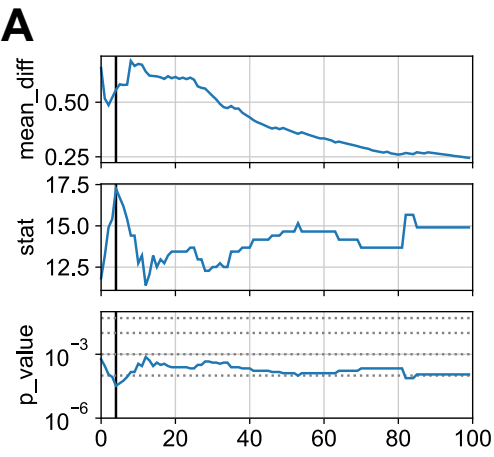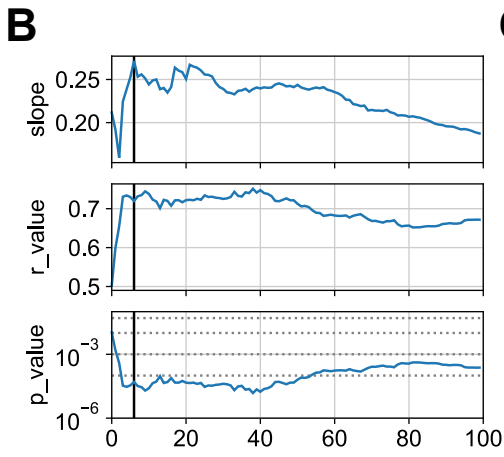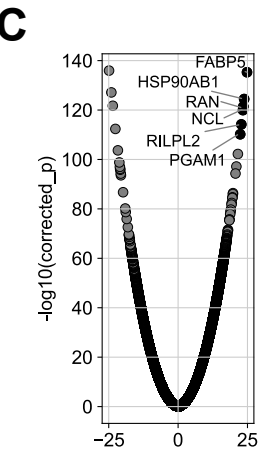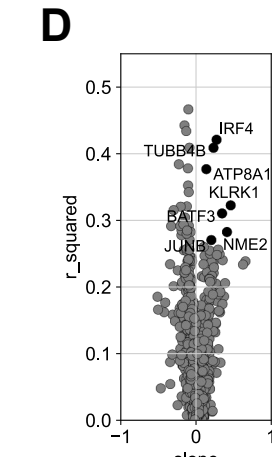

Figure S5

Supplement: Supplementary file 1 [file vaccines-10-01617-s001.zip › Supplementary figures_Mateyka et al.pdf]
